# Development of Models to Estimate Total Soil Carbon across Different Croplands at a Regional Scale Using RGB Photography

**DOI:** 10.3390/ijerph19159344

**Published:** 2022-07-30

**Authors:** Yeon-Kyu Sonn, Jun-Hyuk Yoo, Deogratius Luyima, Jae-Han Lee, Jin-Hyuk Chun, Yun-Gu Kang, Taek-Keun Oh, Jaesung Cho

**Affiliations:** 1 Soils & Fertiliser Management Division, Department of Agricultural Environment, National Institute of Agricultural Science, Rural Development Administration, Wanju 55365, Korea; sonnyk@korea.kr; 2 Department of Bio-Environmental Chemistry, College of Agricultural and Life Sciences, Chungnam National University, Daejeon 34134, Korea; tim2924@naver.com (J.-H.Y.); deoluyima@gmail.com (D.L.); lollolljh@naver.com (J.-H.L.); slaldinv2@gmail.com (J.-H.C.); zserf078@naver.com (Y.-G.K.); 3 Department of Animal Science and Biotechnology, College of Agricultural and Life Sciences, Chungnam National University, Daejeon 34134, Korea

**Keywords:** multiple regression models soil colour, RGB photography, total soil carbon

## Abstract

A quick, accurate and cost-effective method for estimating total soil carbon is necessary for monitoring its levels due to its environmentally and agronomically irreplaceable importance. There are several impediments to both laboratory analysis and spectroscopic sensor technology because the former is both expensive and time-consuming whereas the initial cost of the latter is too high for farmers to afford. RGB photography obtained from digital cameras could be used to quickly and cheaply estimate the total carbon (TC) content of the soil. In this study, we developed models to predict soil TC contents across different cropland types including paddy, upland and orchard fields as well as the TC content of the soil combined from all the aforementioned cropland types on a regional scale. Soil colour measurements were made on samples from the Chungcheongnam-do province of South Korea. The soil TC content ranged from 0.045% to 6.297%. Modelling was performed using multiple linear regression considering the soil moisture levels and illuminance. The best soil TC prediction model came from the upland soil and gave training and validation r^2^ values of 0.536 and 0.591 with RMSE values of 0.712% and 0.441%, respectively. However, the most accurate equation is the one that produces the lowest RMSE value. Hence, although the model for the upland soil was the most stable of all, the paddy soil model which gave training and validation r^2^ values of 0.531 and 0.554 with RMSE values of 0.240% and 0.199%, respectively, was selected as the best soil TC prediction equation of all due to its comparatively high r^2^ value and the lowest RMSE of all equations.

## 1. Introduction

Soil carbon has attracted superabundant interest within the science community in recent decades as revealed by the steady and exponential growth in the number of scientific publications since the early 2000s [1]. The increased interest in soil carbon research partly stems from its already known role of enhancing and or sustaining soil quality and health [2]. Notably, soil carbon especially the organic fraction has been indicated to directly impact soil fertility with incidental influences on plant growth/crop yield, e.g., Zvomuya et al. [3] found that crop yields increased with increasing soil organic carbon (SOC) content until an optimum amount beyond which no more yield increments could be detected. Additionally, SOC influences the soil structure and aggregate stability with implications for soil erodibility while impacts on both water and nutrient retentions, releases and use efficiencies are well documented. However, much of the interest in soil carbon ensues from its potential to mitigate greenhouse gas (GHGs) emissions through sequestering atmospheric carbon in the soil for prolonged periods of time [1].

Indeed, soil is the second largest reservoir of carbon whose storage capacity of more than 6000 Pg of carbon in the uppermost 3 metre depth is only dwarfed by that of the oceans which amounts to about 38,000 Gt of carbon [4]. To a 3 m depth of the soil, therefore, the stock of soil carbon is about 7.5 times that of the atmosphere which amounts to around 800 Pg and approximately 9.7 times that of the biomass whose quantity stands at 620 Pg [4]. Given its sheer importance, both agriculturally and environmentally, it is vital to develop cheap, rapid and accurate methods of monitoring soil carbon levels. For that matter, Wu et al. [5] and Heil et al. [6] recently proposed the use of digital RGB photography for the hasty estimation of soil organic carbon/matter. However, unless taken under fixed lighting conditions, the soil colour measurements are affected by changes in the spectral properties and amount of natural daylight [7].

Additionally, moisture is a very important dynamic variable that affects the tone of soil colour since moist soils tend to be darker than the dry ones [8]. Indeed, Heil et al. [6] demonstrated that both reflectance and soil moisture had effects on the RGB values of the soils. Thus, it is important to include reflectance and moisture measurements in the RGB soil carbon predictive models to cater for the variations in soil colour caused by variations in natural daylight conditions and moisture levels. It is worth noting, however, that both organic and inorganic fractions of the soil carbon are vital since the inorganic fractions actually account for approximately 38% of the global total [9,10], although attention has been hitherto only placed on the organic fraction of the soil. The aim of this study was to develop models that can accurately estimate the contents of the total soil carbon using variables of colour spaces obtained from the images of a smart phone digital camera.

## 2. Materials and Methods

### 2.1. Study Area, Soil Sampling and Processing of the Samples

**Figure 1:**
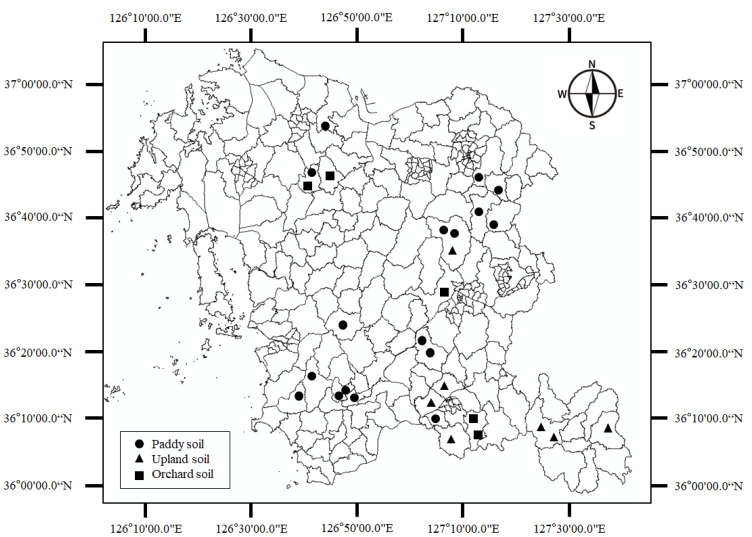
Overview of the study area, Chungcheongnam-do province of South Korea. Locations of the sampling sites are shown as black dots.

The study sites were in the Chungcheongnam-do province of South Korea. The map of the study area is shown in Figure 1. The soil samples were collected from three different types of cropland including orchards, paddy and upland fields. The soils predominantly belong to the Inceptisol class but considerable amounts of Entisols exist, especially along the coast while Ultisols exist only in infinitesimal quantities (the soil class categorisation is based on the USDA soil taxonomy). The soils in this region formed from both metamorphic and acidic rocks and are weakly acidic with pH ranges of between 5.5 and 6.0 (http://soil.rda.go.kr/eng/soils/survey.jsp, accessed on 14 March 2022). The predominant soil texture is sandy loam but clay loam is predominant along the areas abutting the coast. A total of 87 soil cores were picked with an auger from three different agricultural land use types which included paddy fields (51 cores), upland fields (21 cores) and orchard fields (15 cores). The soil was dug to a depth of 70 cm. The auger containing the soil sample was covered tightly with a plastic wrap to prevent the evaporation of water and then taken to the laboratory for further processing.

**Figure 2:**
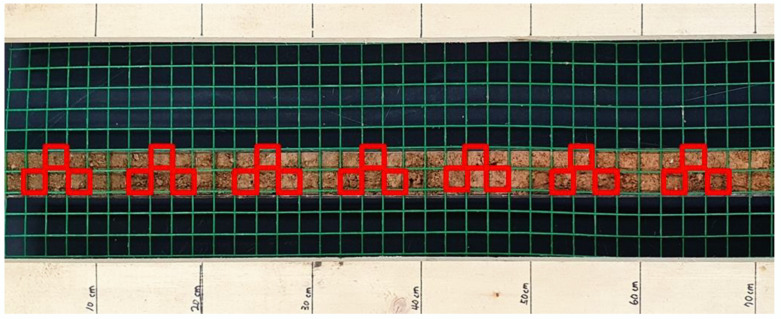
The photograph of the auger with a grid frame on top. The red squares represent the area set for RGB analysis.

A laboratory-made grid frame (with a grid spacing of 2 cm in both length and width) was laid over the soil in the auger. The soil core was divided into seven equal portions of 10 cm length each using the markings made at every 10 cm length of the grid frame and for each 10 cm length, 3 grids were randomly selected and the soil in them was photographed using a Samsung S10 (SM-G973N) phone. The soil in each of the photographed grid was emptied into a crucible dried at 105 °C for 24 h and determined for total carbon with an elemental analyser (TruSpec Micro CHN, LECO). The average carbon content of each of the 10 cm-long dimensions of the soil core was obtained from the individual contents of each of the photographed grid. Therefore, a total of 1827 photographs and soil samples were analysed but the model development was executed using 609 datasets (average values). Consequently, the carbon prediction models of the individual cropland types of paddy, upland and orchard fields were executed with 357, 147 and 105 datasets (average values). Figure 2 is a simple illustration of how RGB values were obtained from the laboratory. Along with the RGB values, the soil illuminance/reflectance values of the photographed samples were obtained using the Samsung S10 lux meter.

### 2.2. Soil Carbon Prediction Model Development Process

#### 2.2.1. Statistical Analysis

The TC content of the soil was set as a dependent variable while the RGB values of the soil, water content, and indoor illuminance (lx) were set as independent variables in the multiple regression analysis. Using Stata (StataCorp. version 16, College Station, TX, USA), descriptive statistics of each of the variables, i.e., TC, RGB, water content and lx including the mean, standard deviation, maximum, and minimum values were obtained. Additionally, the correlation relationships between the RGB values of the soil and each of the soil variables measured in the laboratory including moisture content, lx and total carbon content were assessed. The results of the correlation analysis were evaluated for their possibility to predict the TC content of the soil. There were very high correlations amongst R, G and B which could cause a problem of multicollinearity. Therefore, after the regression analysis, the variance inflation factor (VIF) values of R, G and B were measured to determine the probability of occurrence of multicollinearity. Consequently, since the VIF values measured were greater than 10, it was determined that the probability of the occurrence of multicollinearity was very high and hence, a principal component analysis (PCA) of R, G and B was performed. Three principal components that had no correlations with R, G, and B but reflected all the variances of R, G and B were extracted through the PCA. However, the third principal component was excluded from the regression analysis because its importance/weight was very small. The coefficients were calculated using ordinary least squares after composing the TC contents of the soils as a linear function of the first and second principal components extracted from RGB values as well as the soil moisture content and lx.

#### 2.2.2. Calculation and Validation of Predictive Regression Models

Regression models to predict the TC contents of the soils of each individual cropland types as well as the soil TC content of the of the sum of all the croplands were developed. This was achieved using eigenvectors for each principal component derived from the PCA, estimation coefficients of each principal component, moisture content and lx derived from the multiple regression analysis and standardised values of R, G and B obtained through the colour analysis programme. Finally, the accuracy and reliability of the regression models to predict the TC contents of the soils were verified by comparisons of the TC contents obtained from the CHN analysis to the values of the prediction models. A total of 609 (74.4%) training sets were used to calculate the soil total carbon content from the prediction regression model while 210 (25.6%) validation sets were used for the validation of the results. The training sets used to generate the TC contents from the prediction regression models of the individual cropland types were 357 (73.9%), 147 (70.0%) and 105 (71.4%), respectively, for the paddy, upland and orchard fields, respectively, whilst the validation sets used for validating the results were 126 (26.1%), 63 (30.0%) and 42 (28.6%) for the paddy, upland and orchard fields, respectively. Both the coefficient of determination (r^2^) and root mean square error (RMSE) methods were used to evaluate and validate the calculated TC prediction models.

## 3. Results

### 3.1. Descriptive, Statistical and Model Development Results

The descriptive statistical data are shown in Table S1 and Figure S1 of the Supplementary Materials. The average soil TC content of all the croplands (TS) stood at 0.932% with a standard deviation of 0.776% which was similar to the contents obtained from the paddy soil (PS) whose standard deviation was 0.639%. The lowest average value of total soil carbon was 0.726% obtained from upland soil (US) with a standard deviation of 0.963% which was the highest of all while the orchard soil (OS) contained the highest quantities of total soil carbon, which amounted to 1.219% on average with a standard deviation of 0.823%. The TC content of the US soil exhibited the largest standard deviation amounting to 0.963%, which might have resulted from the maximum value of the total carbon content of US being higher than the maximum values of the rest of the soils. The mean values and standard deviations of R, G, and B of all the soils (TS) and those of the soils of the individual cropland types are shown in Figure S1.

The average values of R for TS, PS, US and OS stood at 151.598, 146.389, 169.517 and 144.219, respectively. The G values of TS, PS, US and OS amounted to 130.920, 131.664, 137.837 and 118.705, respectively. The B values of TS, PS, US and OS stood at 111.580, 113.784, 111.429 and 104.295, respectively. The average soil moisture content of TS PS, US and OS amounted to 22.345%, 22.932%, 17.985% and 26.452, respectively. The average values of lx of TS, PS, US and OS were 299.586 (lm m^−2^), 306.412 (lm m^−2^), 305.000 (lm m^−2^), and 268.800 (lm m^−2^), respectively.

As shown in Table 1, the TC content of TS exhibited negative linear relationships with R (r^2^ = −0.456), G (r^2^ = −0.321) and B (r^2^ = −0.142). Additionally, the T-C of TS exhibited positive (r^2^ = 0.548) and negative (r^2^ = −0.210) relationships with the soil water content (WC) and lx, respectively. The R value exhibited positive relationships with G (r^2^ = 0.891) and B (r^2^ = 0.655). Additionally, the R exhibited positive (r^2^ = 0.382) and negative (r^2^ = −0.440) relationships with lx and WC, respectively. The G value had positive relationships with B (r^2^ = 0.901) and lx (r^2^ = 0.426) but a negative relationship with WC (r^2^ = −0.440). The relationship between B and WC was negative (r^2^ = −0.220) but its relationship with lx was positive (r^2^ = 0.367). The relationship between WC and lx was negative (r^2^ = −0.152).

For the correlation analysis in PS, a total of 357 datasets (53.62%) were used as shown in Table 2. The T-C in PS exhibited negative linear relationships with R (r^2^ = −0.649), G (r^2^ = −0.540) and B (r^2^ = −0.359). The relationship with WC was positive (r^2^ = 0.478) while the one with lx was negative (r^2^ = −0.428). R had positive linear relationships with both G (r^2^ = 0.912) and B (r^2^ = 0.687) while its relationships with WC and lx were negative (r^2^ = −0.442) and positive (r^2^ = 0.368), respectively. Additionally, there was a positive relationship (r^2^ = 0.902) between G and B. The relationship between G and WC was negative (r^2^ = −0.447) whilst the one with lx was positive (r^2^ = 0.35). B exhibited positive (r^2^ = 0.300) and negative and (r^2^ = −0.408) linear relationships with lx and soil moisture, respectively. The linear relationship between WC and lx was negative (r^2^ = −0.203).

For correlation analysis in the US, a total of 147 datasets (average values) were used. As shown in Table 3, T-C in the US exhibited negative linear relationships with R (r^2^ = −0.527), G (r^2^ = −0.397) and B (r^2^ = −0.209). The relationships of T-C with WC and lx were positive with r^2^ = 0.620 and r^2^ = 0.104, respectively. The R value exhibited positive linear relationships with both G (r^2^ = 0.899) and B (r^2^ = 0.637) indicating that there were very high correlations with each other. The relationships of R with WC and lx were negative (r^2^ = −0.370) and positive (r^2^ = 0.039), respectively. The relationships of G with B, WC and lx were r^2^ = 0.879, r^2^ = −0.353 and r^2^ = −0.037, respectively. The relationships of B with WC and lx were negative at r^2^ = −0.290 and −0.211, respectively. WC and lx had a positive linear relationship at r^2^ = 0.172. The results in Table 3 indicate that the variables other than the variable lx were statistically significant at the significance level within 5%.

The correlation analyses in the OS were conducted using a total of 105 datasets (average values), the results of which are presented in Table 4. T-C exhibited positive linear relationships with R (r^2^ = 0.110), G (r^2^ = 0.304) and B (r^2^ = 0.371). Therefore, while the T-C of all the other soils investigated in the study exhibited negative correlation relationships with the RGB, T-C in the OS displayed a positive relationship with RGB. The relationships of T-C with WC and lx were also positive at r^2^ = 0.585 and r^2^ = 0.283, as shown in Table 4. The linear relationships of R with both G and B were positive at r^2^ = 0.924 and r^2^ = 0.755, respectively. Additionally, both negative (r^2^ = −0.316) and positive (r^2^ = 0.828) linear relationships of R with WC and lx, respectively, were observed. G exhibited positive linear relationships with B (r^2^ = 0.941) and lx (r^2^ = 0.879) but a negative relationship with WC (r^2^ = −0.014). The B value exhibited positive relationships with both WC (r^2^ = 0.209) and lx (r^2^ = 0.802). The linear relationship between WC and lx was positive (r^2^ = 0.033) but it was not statistically significant. All the variables in the TS and PS were statistically significant at the *p* < 0.01 significance level but the variables in the US and OS exhibited statistical significance at both the 1% and 5% significance levels while a few variables in both the cropland types did not display any statistical significance. However, since most variables were statistically significant in all soils, the conclusion is that it was viable to predict the total soil carbon content from the models developed from the obtained results.

**Figure 3:**
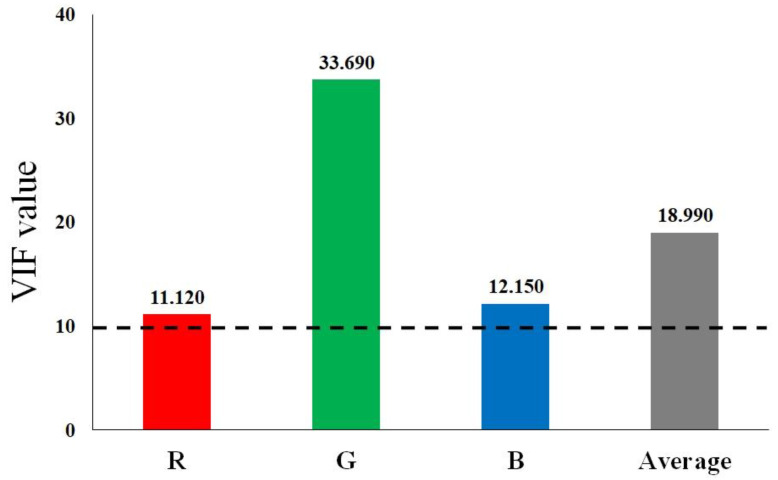
Variance inflation factor results of the RGB.

The possibility of the existence of multicollinearity was adjudged to be high due to the exceedingly high correlations among the R, G and B values. When multicollinearity occurs, the variance of the regression coefficient increases which results in the estimate of the regression coefficient being unstable, and thus, it becomes difficult to interpret the results. As shown in Figure 3, the VIF values of R, G and B were 11.120, 33.690 and 12.150, respectively, with an average value of 18.990 and hence, the probability of the occurrence of multicollinearity was confirmed to be high. Consequently, the components that did not correlate with each other amongst the R, G and B values but reflected the variances of R, G and B were extracted through the PCA. Amongst the three principal components extracted, the first principal component (component 1) and the second principal component (component 2) were found to explain 99.4% of the total variance, as shown in Table 5. Therefore, the third principal component (component 3) was excluded from the regression analysis because its weight (importance) was too insignificant.

Additionally, the eigenvectors of R, G and B which indicate the directions of the main axes to which the calculated principal components data are headed were calculated and are shown in Table 6. The results of the multiple regression analysis performed using the least squares method are shown in Table 7. In the estimated coefficients, components 1 and 2 were calculated as coefficients of the independent variables in the calculated TC prediction models. The regression coefficients of the WC content and lx were calculated as constants reflecting the elasticity of the TC content of the soil together with the constants. The TC prediction models of TS, US, PS and OS developed from the 609 datasets (100%), 357 datasets (53.62%), 147 datasets (24.14%) and 105 datasets (17.24%) are given in Equations (1)–(4), respectively.

T-C = (−0.008R) + (−0.001G) + 0.008B + 0.807 (1)

T-C = (−0.012R) + (−0.004G) + 0.009B + 2.164 (2)

T-C = (−0.013R) + (−0.002G) + 0.021B + 0.905(3)

T-C = 0.007R + 0.003G + (−0.003B) + 0.165(4)

The eigenvectors for each principal component used in the equations are shown in Table 6, and the estimated coefficients are given in Table 7 while the standardised values of R, G and B are presented in Figure S1 of the Supplementary Materials.

### 3.2. Validation of the Datasets

The datasets of the TC content obtained from the elemental analyser were used to verify the accuracy and reliability of the regression models given in Equations (1)–(4) developed for the predictions of the soil TC content. As seen from Table 8, the r^2^ values of the training and validation sets of the first equation (TS) were 0.391 and 0.453, respectively. Therefore, since the validation sets showed a higher coefficient of determination than the training sets, it was confirmed that the first equation was relatively stable in predicting the soil TC content. The RMSE values of the training and validation sets of the first equation (TS) were 0.810 and 0.652. It was thus confirmed that since the validation sets produced a lower RMSE value than the training sets, the first equation was proven to be relatively accurate in predicting the TC content of the soil.

The r^2^ values of the training and validation sets of the PS equation (Equation (2)) were 0.531 and 0.554, respectively as shown in Table 8. Hence, the second equation was judged to have a higher stability in predicting the soil TC content than Equation (1) since the former produced higher coefficients of determination for both training and validation sets than the latter. On the other hand, the RMSE values of the training and validation sets of the PS (Equation (2)) were 0.240 and 0.199, respectively. This proves the accuracy of Equation (2) since the validation sets showed a lower RMSE value than the training sets, although equation one is more accurate due to the higher RMSE values of its training and validation sets.

The r^2^ values of the training and validation sets for the US (Equation (3)) were 0.536 and 0.591, respectively as seen from Table 8. As was the case for Equations (1) and (2), the reliability/stability of 3 was proven because the validation sets showed a higher coefficient of determination than the training sets. The RMSE values of the training and validation sets were 0.712 and 0.441, respectively. Therefore, the accuracy of Equation (3) to predict soil TC content was proven since the validation sets produced a lower RMSE value than the training sets. The r^2^ values of the training and validation sets of the OS were 0.448 and 0.506, respectively. The reliability/stability of Equation (4) was also proven since the validation sets showed a higher coefficient of determination than the training sets. The RMSE values of the training and validation sets were 0.725 and 0.503, respectively. Therefore, the accuracy of Equation (4) was proven since the validation sets showed a lower RMSE value than the training sets. The predicted values obtained from all equations except Equation (1) were comparable to the measured values for both the training and validation sets, as can be seen in Table S2 of the Supplementary Materials. This signifies the accuracy and stability/reliability of all the models developed (except model 1) to predict the TC content of the soil.

## 4. Discussions

RGB has been examined for its potential for the simple, quick and accurate analysis of different soil properties. For example, Kumar et al. [11] used RGB to predict soil pH through calculating the pH indices of each of the pixels whose average gives the pH value of the sample. Another study by Aitkenhead et al. [12] indicated that RGB photography gave a good estimation (r^2^ ≥ 0.8) for exchangeable hydrogen but the estimate for organic carbon (loss on ignition) was fair with an r^2^ value of 0.6–0.8. However, as mentioned earlier, RGB values are affected by several factors such as soil moisture levels and reflectance/illuminance. The latter problem ensues from the fact that a digital camera is different from a human eye, and as such, the same object can display various colours in different light intensities [5,13]. It is against this backdrop that this study incorporated both variations in soil moisture and indoor illuminance into the TC prediction equations development process so that the resulting equations are standardised for the prediction of the TC content of the soil.

Even though all the obtained equations were conformed to be stable and accurate in predicting the soil TC content as judged from the coefficients of determination (r^2^) and root mean square error (RMSE) [5] of both the training and validation datasets, the obtained values were low. The best soil TC prediction model gave training and validation r^2^ values at 0.536 and 0.591 with RMSE values of 0.712 and 0.441%, respectively. However, the most accurate equation is the one that produces the lowest RMSE value. Hence, although Equation (3) was the most stable of all, Equation (2) which gave training and validation r^2^ values at 0.531 and 0.554 with RMSE values of 0.240 and 0.199%, respectively, was selected as the best soil TC prediction equation of all due to its comparatively high r^2^ value and lowest RMSE of all the equations. This study, therefore, demonstrates that RGB photography from a digital camera can estimate the soil TC content using the equations developed in this study but the best results are obtainable when estimating TC contents of the paddy soils (Equation (2)).

While the obtained results may seem low, they fall within the range of 0.31–0.99 with a median of 0.80 and 0.1–10.2% with a median of 0.5% reported for R^2^ and RMSE reported by Rossel et al. [14] in a review of 97 studies of the model validations. The results were also comparable to those obtained by Heil et al. [6] who indicated that the best multiple linear regression and random forest models gave validation r^2^ values for the prediction of soil organic carbon at 0.66 and 0.63 with RMSE values of 0.57 and 0.61%, respectively. Thus, it is concluded that the equations/models developed for the prediction of the soil TC content can give sufficiently good results on the regional scale. This study was conducted on a regional scale with both top and subsoils and hence, geographic factors potentially masked the effect of the TC content of the soil but still soil colour could explain the TC content by approximately 60%. It is therefore expected that local scale calibration models will produce better estimations as Heil et al. [6] noted. Models intended to predict the soil TC content at a regional scale could be improved by incorporating spatial/environmental covariates. Additionally, Heil et al. [6] opined that a large sample set could improve the predictive performance of the models developed which was confirmed in this study because the PS with the largest sample size produced the best prediction model. Another important observation is that mixing soils from different cropland types adversely affects the performance of the prediction models and hence, models could be improved using soils from similar cropland types.

## 5. Conclusions

This study developed models that can predict total soil carbon contents of the soils of different cropland types using the inexpensive smart phone RGB photography by incorporating both the soil moisture content and illuminance into the multiple regression analysis. This is because both the illuminance and moisture contents of the soil have direct effects on the obtained RGB values. The best models obtained using soil colour (RGB) in this study could explain the soil total carbon content by approximately 60% and were comparable to the validation results of several previous studies. The relatively low training and validation r^2^ values obtained might have ensued from mixing top and subsoils or because the sampling was performed at a regional scale. This is because with increasing the scale of sampling, parameters other than soil TC can influence the soil colour and thus, weaken the performance of the prediction models. Bearing that in mind, therefore, the prediction models developed in this study were reasonable. However, the prediction could be improved by working on a smaller scale or possibly by incorporating spatial/environmental covariates into the models.

## Figures and Tables

**Table 1:** Pearson correlation coefficient table of the TS soil components.

	TC	R	G	B	WC	lx
**TC**	1.000					
**R**	−0.456 ***	1.000				
**G**	−0.321 ***	0.891 ***	1.000			
**B**	−0.142 ***	0.655 ***	0.901 ***	1.000		
**WC**	0.548 ***	−0.440 ***	−0.358 ***	−0.220 ***	1.000	
**lx**	−0.210 ***	0.382 ***	0.426 ***	0.367 ***	−0.152 ***	1.000

***, ** and * indicate statistical significance at the 1%, 5% and 10% significance levels, respectively. WC: water content of the soil; lx: soil illuminance; TC: total carbon content of the soil.

**Table 2:** Pearson correlation coefficient table of the PS soil components.

	T-C	R	G	B	WC	lx
**T-C**	1.000					
**R**	−0.649 ***	1.000				
**G**	−0.540 ***	0.912 ***	1.000			
**B**	−0.359 ***	0.687 ***	0.902 ***	1.000		
**WC**	0.478 ***	−0.442 ***	−0.447 ***	−0.408 ***	1.000	
**lx**	−0.428 ***	0.368 ***	0.351 ***	0.300 ***	−0.203 ***	1.000

***, ** and * indicate statistical significance at 1%, 5% and 10% significance levels, respectively. WC: water content of the soil; lx: soil illuminance; TC: total carbon content of the soil.

**Table 3:** Pearson correlation coefficient table of the US soil components.

	T-C	R	G	B	WC	lx
**T-C**	1.000					
**R**	−0.527 ***	1.000				
**G**	−0.397 ***	0.899 ***	1.000			
**B**	−0.209 **	0.637 ***	0.879 ***	1.000		
**WC**	0.620 ***	−0.370 ***	−0.353 ***	−0.290 ***	1.000	
**lx**	0.104	0.039	−0.037	−0.211 **	0.172 **	1.000

***, ** and * indicate statistical significance at 1%, 5% and 10% significance levels, respectively. WC: water content of the soil; lx: soil illuminance; TC: total carbon content of the soil.

**Table 4:** Pearson correlation coefficient table of the OS soil components.

	T-C	R	G	B	WC	lx
**T-C**	1.000					
**R**	0.110	1.000				
**G**	0.304 ***	0.924 ***	1.000			
**B**	0.371 ***	0.755 ***	0.941 ***	1.000		
**WC**	0.585 ***	−0.316 ***	−0.014	0.209 **	1.000	
**lx**	0.283 ***	0.828 ***	0.879 ***	0.802 ***	0.033	1.000

***, ** and * indicate the statistical significance at 1%, 5% and 10% significance levels, respectively. WC: water content of the soil; lx: soil illuminance; TC: total carbon content of the soil.

**Table 5:** Eigenvalues derived from the principle component analysis.

	Eigenvalue	Proportion	Cumulative
**Component 1**	2.636	0.879	0.879
**Component 2**	0.345	0.115	0.994
**Component 3**	0.019	0.006	1.000

**Table 6:** Eigenvectors derived from the principle component analysis.

	Component 1	Component 2	Component 3
**R**	0.558	0.715	0.421
**G**	0.612	−0.012	−0.791
**B**	0.560	−0.699	0.445

**Table 7:** Multiple regression analysis results obtained using ordinary least squares.

	Classification	Estimation Coefficient	Robust Standard Error	t-Value	*p* > |t|
**Component 1**	TS	−0.068	0.020	−3.420	0.001
	PS	−0.162	0.018	−9.060	0.000
	US	−0.070	0.060	−1.160	0.246
	OS	0.166	0.068	2.440	0.016
**Component 2**	TS	−0.353	0.042	−8.450	0.000
	PS	−0.425	0.040	−10.590	0.000
	US	−0.630	0.167	−3.760	0.000
	OS	0.277	0.197	1.410	0.162
**WC**	TS	0.040	0.006	6.540	0.000
	PS	0.022	0.005	4.100	0.000
	US	0.076	0.024	3.110	0.002
	OS	0.066	0.014	4.890	0.000
**lx**	TS	−0.001	0.000	−2.660	0.008
	PS	−0.002	0.000	−5.340	0.000
	US	0.003	0.001	2.260	0.026
	OS	−0.001	0.002	−0.470	0.642
**Constant**	TS	0.317	0.182	1.740	0.083
	PS	0.891	0.181	4.930	0.000
	US	0.671	0.275	−4.960	0.000
	OS	0.943	0.652	−0.300	0.768

WC: water content of the soil; lx: soil illuminance.

**Table 8:** Validation results of the soil TC content prediction models.

Equations	Parameter	R^2^ Value	RMSE Value
Equation (1)	Training	0.391	0.810
	Validation	0.453	0.652
Equation (2)	Training	0.531	0.240
	Validation	0.554	0.199
Equation (3)	Training	0.536	0.712
	Validation	0.591	0.441
Equation (4)	Training	0.448	0.725
	Validation	0.506	0.503

## Data Availability

The data supporting the conclusions made in this study are included in the article.

## Supplementary Materials

The following supporting information can be downloaded at: https://www.mdpi.com/article/10.3390/ijerph19159344/s1, Figure S1: Average TC (a), water content (b), illuminance (c), R (d), G (e) and B (f) values of the soils of the different crop land types; Table S1: Basic statistics (minimum and maximum values) used in multiple regression analysis; Table S2: The measured and predicted values of both the training and validation datasets of the different crop land types.
