# Supplementary material for: Development of Models to Estimate Total Soil Carbon across Different Croplands at a Regional Scale Using RGB Photography
Source: Int J Environ Res Public Health. 2022 Jul 30;19(15):9344. doi: 10.3390/ijerph19159344 (PMC9368161; doi:10.3390/ijerph19159344)
Supplement: Supplementary file 1 [file ijerph-19-09344-s001.zip › ijerph-1823091-supplementary.pdf]

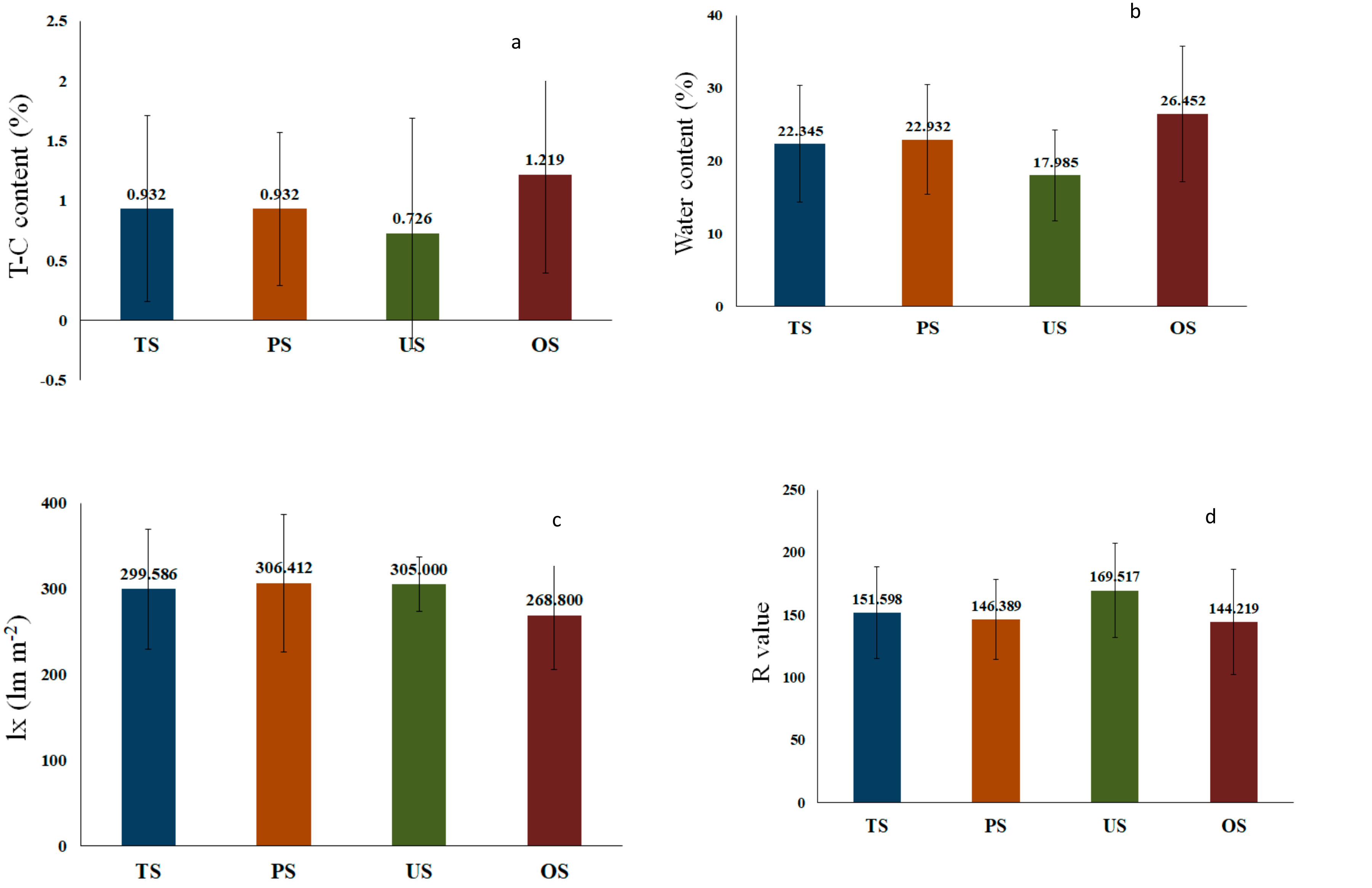
a
T-C content (%)
2.5
2
1.5
1
0.5
0
-0.5
0.932
0.932
0.726
1.219
TS
PS
US
OS
b
Water content (%)
40
30
20
10
0
22.345
22.932
17.985
26.452
TS
PS
US
OS
c
lx (lm m-2)
400
300
200
100
0
299.586
306.412
305.000
268.800
TS
PS
US
OS
d
R value
250
200
150
100
50
0
151.598
146.389
169.517
144.219
TS
PS
US
OS

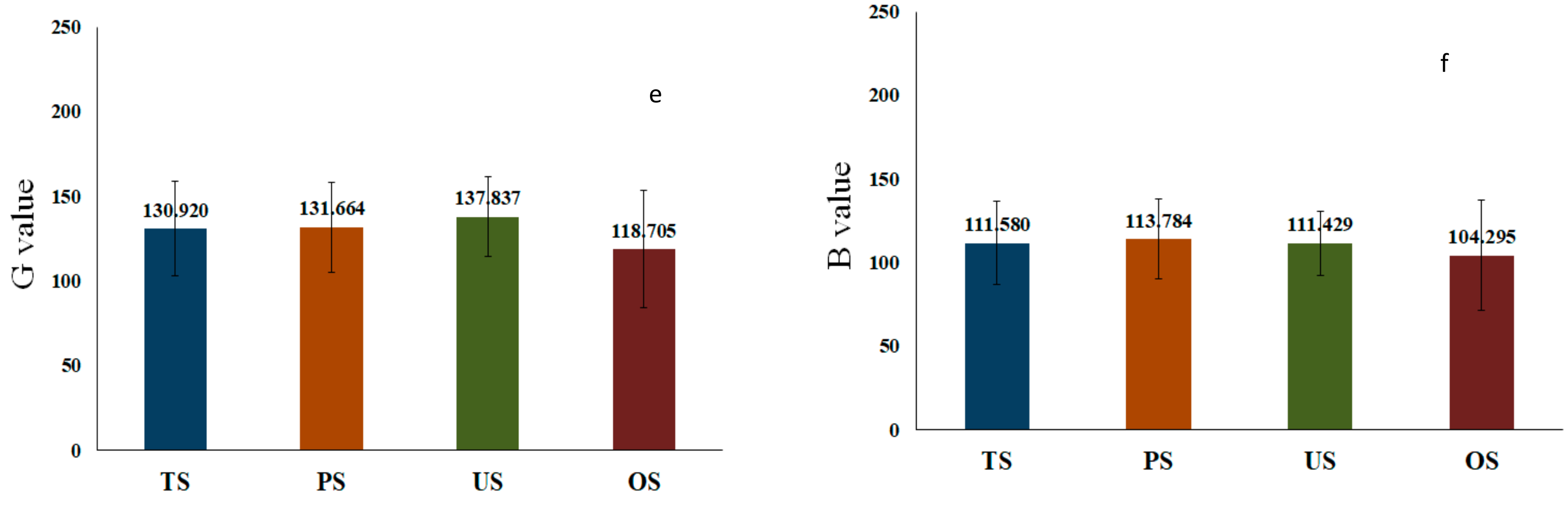

Figure S1. Average TC (a), water content (b), illuminance (c), R (d), G (e) and B (f) values of the soils of the different crop land types.

Table S1. Basic statistics (minimum and maximum values) used in multiple regression analysis.

	**Classification**	**T-C (%)**	**R (red)**	**G (green)**	**B (blue)**	**WC (%)**	**lx (lm m^{-2})**
**Minimum value**	TS (n=609)	0.045	47.000	37.000	31.000	8.146	145.000
	PS (n=357)	0.047	54.000	50.000	49.000	8.146	182.00
	US (n=147)	0.045	74.000	74.000	69.000	9.295	263.000
	OS (n=105)	0.134	47.000	37.000	31.000	12.540	145.000
**Maximum value**	TS (n=609)	6.297	241.00	219.000	197.000	50.013	405.000
	PS (n=357)	4.576	238.000	219.000	197.000	49.614	405.000
	US (n=147)	6.297	241.000	188.000	163.000	45.134	376.000
	OS (n=105)	3.758	206.000	176.000	163.000	50.013	309.000

WC; Water Content, TS; soil of all the crop lands, PS; Paddy soil, US, Upland Soil, OS; Orchard Soil, lx; soil illuminance.

Table S2. The measured and predicted values of both the training and validation datasets of the different crop land types.

	**Training sets**		**Validation sets**	
	**MV**	**PV**	**MV**	**PV**
**TS**	0.932±0.776	0.356±0.223	1.044±0.610	0.420±0.164
**PS**	0.932±0.639	0.905±0.351	0.970±0.542	0.885±0.259
**US**	0.726±0.963	0.766±0.402	0.432±0.184	0.830±0.288
**OS**	1.129±0.823	1.128±0.316	0.821±0.733	0.691±0.149

Abbreviations: MV; measured value, PV; predicted value, TS; soil of all the crop lands, PS; Paddy soil, US, Upland Soil, OS; Orchard Soil
